# mTOR as a Marker of Exercise and Fatigue in *Octopus vulgaris* Arm

**DOI:** 10.3389/fphys.2019.01161

**Published:** 2019-09-11

**Authors:** Federica Maiole, Sarah Giachero, Sara Maria Fossati, Anna Rocchi, Letizia Zullo

**Affiliations:** ^1^Center for Synaptic Neuroscience and Technology, Istituto Italiano di Tecnologia, Genoa, Italy; ^2^Department of Experimental Medicine, University of Genoa, Genoa, Italy; ^3^IRCSS Ospedale Policlinico San Martino, Genoa, Italy

**Keywords:** mTOR, cephalopods, muscle, metabolism, welfare, growth, exercise

## Abstract

Cephalopods are highly evolved marine invertebrates that colonized almost all the oceans of the world at all depths. This imposed the occurrence of several modifications of their brain and body whose muscle component represents the major constituent. Hence, studying their muscle physiology may give important hints in the context of animal biology and environmental adaptability. One major pathway involved in muscle metabolism in vertebrates is the evolutionary conserved mTOR-signaling cascade; however, its role in cephalopods has never been elucidated. mTOR is regulating cell growth and homeostasis in response to a wide range of cues such as nutrient availability, body temperature and locomotion. It forms two functionally heteromeric complexes, mTORC1 and mTORC2. mTORC1 regulates protein synthesis and degradation and, in skeletal muscles, its activation upon exercise induces muscle growth. In this work, we characterized Octopus vulgaris mTOR full sequence and functional domains; we found a high level of homology with vertebrates’ mTOR and the conservation of Ser^2448^ phosphorylation site required for mTORC1 activation. We then designed and tested an *in vitro* protocol of resistance exercise (RE) inducing fatigue in arm samples. We showed that, upon the establishment of fatigue, a transient increase in mTORC1 phosphorylation reaching a pick 30 min after exercise was induced. Our data indicate the activation of mTORC1 pathway in exercise paradigm and possibly in the regulation of energy homeostasis in octopus and suggest that mTORC1 activity can be used to monitor animal response to changes in physiological and ecological conditions and, more in general, the animal welfare.

## Introduction

Cephalopods are an important component of marine ecosystems, they count around 800 living marine species, and reached over time unprecedented environmental adaptability. They are not only abundant but also ecologically and economically important (Boyle and Rodhouse, 2005). Their incredible evolutionary success and high adaptability to extreme environments such as the polar and tropical climates and even the deep ocean have been possible through modifications at several levels of their biological organization, from genome to nervous system and body organization (Garrett and Rosenthal, 2012; Nakajima et al., 2018). Nonetheless, a high degree of conservation of regulatory genes has been recently assessed among cephalopods, vertebrates, insects, and other marine invertebrates (Albertin et al., 2012).

Cephalopods have large, well-developed brains, and their brain-to-body-mass ratio exceeds that of all the other invertebrates. Muscles represent their major body constituent and, octopuses in particular, have a rapid growth rate reaching up to 5% body weight per day and manifest a high feed conversion rate, with 30–60% of ingested food being incorporated in their own weight (Iglesias et al., 2000; Aguado and García García, 2002; García García and Cerezo Valverde, 2006). Based on these aspects, studying muscle metabolism in this species can answer important questions relative to the animal biology of growth and welfare.

Several investigations have been carried out so far to elucidate the bases of their muscle metabolism and energy consumption and its relation to environmental condition (Seibel and Carlini, 2001; Villanueva et al., 2017). Yet, little is known about specific metabolic pathways involved in processes such as growth, aging, and environmental adaptability.

Similar to vertebrates, muscles of their body are typically striated and for several functional aspects, they can be assimilated to skeletal muscle fibers (Zullo et al., 2017). In vertebrates, the skeletal muscle mass is regulated by a fine equilibrium between anabolism and catabolism, which determines the rate of protein synthesis and degradation as well as muscle fiber size (Schiaffino et al., 2013). An unbalance between anabolic and catabolic pathways leads to atrophy of muscle fibers, when protein degradation exceeds synthesis rate, and to hypertrophy when new protein synthesis is induced.

Several signaling pathways, including the evolutionary conserved mTOR signaling cascade, maintain this process. mTOR is serine/threonine protein kinase that exists in two functionally distinct protein complexes, the rapamycin-sensitive mTOR complex 1 (mTORC1) and the rapamycin-insensitive mTOR complex 2 (mTORC2), that are defined by the presence of Raptor (regulatory protein associated with mTOR) and Rictor (rapamycin-insensitive companion of mTOR) (Saxton and Sabatini, 2017).

mTORC1 plays a critical role in muscle homeostasis because it senses and integrates a broad range of cellular signals from growth factors, hormones, cytokines, amino-acid availability, cellular energy levels and muscle activity (Zoncu et al., 2011). Genetic and pharmacological studies have shown that mTORC1 is required to maintain muscle mass through the regulation of protein translation and autophagy process (Yoon, 2017).

A number of studies have also reported that resistance exercise (RE)-mediated hypertrophy requires mTORC1 activation, mainly through the phosphorylation of mTOR at serine 2448 (Ser^2448^) (Nave et al., 1999; Chiang and Abraham, 2005; Copp et al., 2009). The resulting increase in myofibrillar protein synthesis and muscle mass takes place by the integration of multiple signaling pathways that are able to control and modulate transcription factors and in turn protein synthesis and degradation.

In this work, for the first time in a cephalopod, we investigated the conservation of mTOR and its role during a particular class of training such as the RE inducing muscle fatigue. Muscle fatigue is defined as the inability of muscles to maintain the required power output. Upon establishment of fatigue, muscle contractile force shows a rapid decline reversible by rest. We tested samples of arm with a specifically designed RE and showed the induction of a fatigue state. We next assessed mTOR pathway following tetanization and show a sharp increase in its activation state and its time dependency.

We believe that studying mTOR pathway may be important to understand the ability of muscles, over time, to adapt to various physiological and ecological conditions such as exercise response, metabolic stress and nutrient availability and may prompt to the development of advanced methods for monitoring cephalopod welfare.

## Materials and Methods

### Animals Treatment

Specimens of *O. vulgaris* were collected from local anglers of the Ligurian coast. Adult animals of both sexes (*n* = 9) ranging between 150 and 250 g and not showing signs of damage and/or regeneration of the arms employed (L2 or L3) were selected for this study.

Following captures, the animals were placed in 80 × 50 × 45 cm marine aquarium tanks filled with artificially prepared sea water (SW, Tropic Marine) and kept at a temperature of ∼18°C at 12 h light/dark cycle. Octopus environment was enriched with sand substrate and clay pot dens. Water cleaning and oxygenation were assured by a pump-filter and aeration system which continuously circulated the water through biological filters; all relevant chemo/physical water parameters were checked daily to prevent unhealthy or stressful conditions for the animals. Animals were left to adapt to captivity for at least 10 days before experimentation. Octopuses were inspected daily and fed with shrimps 3 times per week. Particular attention was paid to housing, animal care, and health monitoring. All our research conformed to the ethical principles of the three Rs (replacement, reduction and refinement) and of minimizing animal suffering, following the Directive 2010/63/EU (Italian D. Lgs. n. 26/2014) and the guidelines from Fiorito et al. (2015).

For molecular biology experiments, 3 animals were anesthetized in ethanol 2% (v/v) in SW. Brain and arm samples (from L2 or L3), devoid of skin and suckers, were collected, frozen in liquid nitrogen and immediately stored at −80°C. Brain samples (from the supraoesophageal mass) were employed as an additional control in western blotting experiments.

For biomechanical experiments a total of 6 animals were anesthetized in 3.5% MgCl_2_ in SW, since ethanol exposure induced muscle stiffness. After anesthesia, a single segment (∼4–5 cm) per animal was cut from the middle-end of the L2 or L3 arm. Arm samples were moved to ∼18°C oxygenated artificial sea water (ASW) (pH 7.6) containing: NaCl, 460 mM; KCl, l10 mM; MgCl_2_, 55 mM; CaCl_2_, 11 mM; Hepes, 10 mM; glucose 10 mM. This temperature was the same as that of the aquarium where the animals were maintained and was within the temperature range of the Mediterranean sea.

Given the large portion of arm excised and/or the dissection of the brain, the animals underwent terminal anesthesia in order to prevent animal suffering or distress and following the Guidelines for the Care and Welfare of Cephalopods in Research published by Fiorito et al. (Fiorito et al., 2014, 2015).

### Ethics Statement

This study was carried out in accordance with the recommendations of Fiorito et al. (Fiorito et al., 2014, 2015). The protocol was approved by the Institutional Review Board and by the Italian Ministry of Health (authorization no. 465/2017-PR).

### RNA Preparation and Sequencing

Total RNA has been extracted from octopus arm segments (*n* = 3) using RNeasy Microarray Tissue Mini Kit (Qiagen) and contaminating DNA has been degraded by treating each sample with RNase-Free DNase Set (Qiagen). The purity of total RNA extracted has been estimated measuring 260/280 and 260/230 absorbance ratios. For each sample, 1 μg of total RNA extracted have been retrotranscribed with ImProm-II(TM) Reverse Transcription System (Promega) following the manufacturer’s instructions. mTOR gene has been divided into small fragments up to 1.5 kb and the correspondent primers were designed (Supplementary Table S1). 2 μL of Octopus cDNA was used as a template for PCR. Fragments obtained from PCR have been purified and sequenced using the automated Sanger method (Applied Biosystems 3130 DNA Analyzer). The identity of segments has been verified and analyzed by BLASTX and BLASTP programs. Consensus sequences were obtained from fragment overlap. Nucleotide and protein sequences were aligned with the human mTOR using Clustal Omega and protein similarities have been calculated using BLASTP at NCBI Genbank. Protein domain annotation and conservation level were assessed by NCBI Conserved Domain Database. Molecular weight was predicted from the amino acid sequence using the ExPASy Compute pI/Mw tool.

### Resistance Exercise Protocol

Muscle fatigue was investigated *in vitro* on total arm segments (*n* = 6) using a Dual-Mode Lever Arm System (Aurora Scientific – 300C-LR) mounted on a muscle test chamber with integrated stimulating electrodes (Aurora Scientific – 801C). Field stimulation was delivered through a current-voltage bi-phase stimulator (Aurora Scientific – 701B). Data were acquired at a sampling frequency of 10 kHz, bandwidth filtered at 10 Hz - 3.3 kHz and further analyzed with a LabVIEW based Data Acquisition and Analysis System (Aurora Scientific – 604A and 605A). Test chamber was bath filled with temperature-regulated ASW continuously circulating at ∼18°C. Arm samples were placed in the chamber, tied tightly at the force transducer system and allowed to rest for about 5 min before starting the protocol. Recordings were made in isometric condition, thus enabling measurements of the force developed over time upon electrical stimulation of the sample. Muscle length was adjusted such that a transient passive force could be visualized (Milligan et al., 1997; Thompson et al., 2014). Samples were tested for their response to a RE protocol consisting of three tetanic stimulation (pulse width: 0.2 ms negative; pulse frequency: 100 Hz; train duration: 3 s) interspersed with 4 s rest each, repeated 7 times with a rest of 120 s between each repetition. After tetanization, samples were prepared for western blotting. In details each sample, following removal of skin and sucker, was cut in 4 equal segments and maintained in oxygenated ASW at ∼18°C for four different time points (0, 30, 60, and 120 min). A small arm sample, not undergoing RE, was also cut from each arm and used as control (−/−) of western blotting. All samples were then frozen in liquid nitrogen and immediately stored at −80°C.

### Protein Extraction and Western Blotting Analysis

To obtain total lysate, 10–20 mg of brain and arm tissue (*n* = 4 for each experimental group), have been homogenized with TissueLyser (Qiagen) in 600 μL of extraction buffer (1% NP40 – 1% SDS – 50 mM Tris-HCl pH 7,6 – 150 mM NaCl) with protease inhibitor (Complete EDTA-free protease inhibitors, Roche) and phosphatase inhibitor cocktails (Sigma-Aldrich). After incubation for 5 min at 100°C, samples have been sonicated and centrifuged at 4°C for 15 min at 13000 × rpm to remove cell debris. The soluble fraction was collected, and protein concentration was determined using the bicinchoninic acid (BCA) assay method (Thermo Scientific). For Western blotting, 80 μg of tissue lysates were boiled in 5X sample buffer (62.5 mM Tris-HCl, pH 6.8, 2% SDS, 25% glycerol, 0.05% bromophenol blue, 5% β-mercaptoethanol, deionized water), resolved by SDS-polyacrylamide gels (SDS-PAGE) and transferred onto nitrocellulose membranes (GE Healthcare BioSciences; 0.20 μm pore size). Mouse gastrocnemius muscle lysates were used as control samples (Rocchi et al., 2016). The following antibodies were used: polyclonal β-actin (A2066, Sigma-Aldrich – 1:50000) and phospho- Ser 2448-mTOR (2971, Cell Signaling Technology – 1:5000). Antibodies were chosen on the basis of the sequence similarity with respective rabbit and human epitopes used to develop the antibodies. Incubation in ECL substrate chemiluminescent detection reagent (GE Healthcare BioSciences) was performed for 1 min at room temperature. The chemiluminescent blots were imaged with the ChemiDoc MP Imaging System (GE Healthcare BioSciences). The Band Analysis tools of ImageLab software version 5.2.1 (Bio-Rad) were used to select and determine the density of the bands in all the blots.

### Statistical Analysis

The software SigmaPlot 13.0 (Systat Software, Inc.) was used for statistical analysis. Normality of the dataset was first assessed with a normality test (Shapiro Wilk). All the dataset analyzed were not normally distributed hence non-parametric test were employed in further analysis. In particular, non-parametric Mann-Whitney Rank Sum Test was used to compare two data sets. Multiple comparisons were performed with Kruskal-Wallis One Way Analysis of Variance (Holm-Sidak correction). *P*-values <0.05 were considered significant.

## Results

### Sequencing of *Octopus vulgaris* mTOR

**FIGURE 1:**
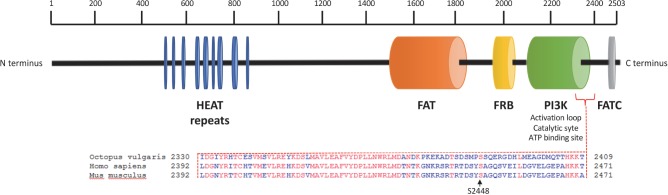
*Octopus vulgaris* mTOR domain architecture and protein alignment in the region of the conserved human and mouse phosphorylation site at Ser2448. *Octopus vulgaris* mTOR full sequence contains about 2503 amino acids; the N-terminus comprises 9 HEAT repeat motifs (blue) followed by a FAT domain (orange). Downstream, mTOR includes a FKPB12-rapamycin binding (FRB) domain (yellow). The C-terminal contains a catalytic domain PI3K (green), with the mTORC1 activation site at Ser^2386^ (conserved between octopus, human and mouse genomes), and a FATC domain (gray).

We sequenced arm samples (*n* = 3) and obtained a single *O. vulgaris* mTOR full-length cDNA sequence consisting of 7629 bp that includes 117 bp of the 5′UTR and an open reading frame (ORF) of 7512 bp encoding 2503 amino acid residues (Figure 1) with a predicted molecular weight of 284.5 kDa. The sequence has been submitted to NCBI under the GenBank Accession No. KY774846. mTOR analysis revealed it as a large multidomain protein and a member of the phosphatidylinositol kinase family. All vertebrate mTOR domains were found to be conserved (Figure 1). HEAT domain at the N-terminus, which mediates protein-protein interaction, FAT domain that binds DEPTOR, an inhibitor of mTOR, FKBP12-rapamycin binding (FRB) which is the target for rapamycin, a macrolide that inhibits mTOR signaling pathway. The catalytic function is mediated by PI3Kinase domain which contains the phosphorylation sites that activate the protein and FAT C-terminal (FATC) domain, at the COOH-terminus. Protein BLAST revealed that S2448 and S2481 residues, whose phosphorylation is respectively involved in mTORC1 and mTORC2 activation (Chiang and Abraham, 2005; Copp et al., 2009), are conserved. Note that S2448 position of human and mouse correspond to S2386 residues in *O. vulgaris* mTOR protein sequence.

### Induction of Muscle Fatigue

**FIGURE 2:**
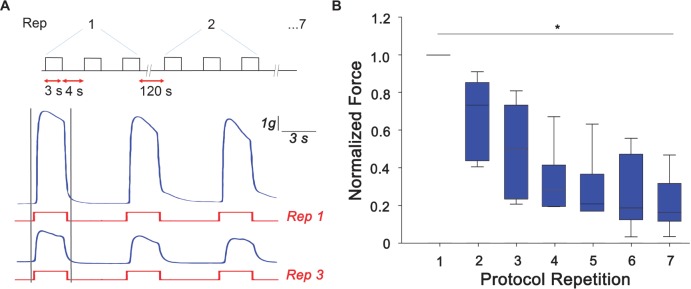
Muscle fatigue induction. **(A)** Resistance exercise (RE) protocol designed to induce muscle fatigue response consisting of three tetanic stimulation (pulse width: 0.2 ms negative; pulse frequency: 100 Hz; train duration: 3 s), 4 s rest each, repeated 7 times with a rest of 120 s. Exemplary traces of force produced (in blue) at the first (Rep 1) and third (Rep 3) stimulus (in red). Peak tetanic force has been analyzed for the first tetanus of each repetition (highlighted with gray bars). **(B)** Peak tetanic force, normalized to the maximum peak tetanic force developed by each sample, recorded during the first tetanus of each repetition (Kruskal-Wallis One Way Analysis of Variance, ^∗^*P* < 0.05; *n* = 6).

Arm samples (*n* = 6) were tested for their ability to develop peak tetanic tension under the designed RE protocol. As a control, arms that did not undergo RE were also tested for their ability to produce peak tetanic tension and no difference was observed over the experimental time scale. Typical traces of samples undergoing RE are shown in Figure 2A where a clear reduction of the peak tetanic tension is observed already at the third repetition of the protocol. Peak forces developed during the first tetanic stimulation of each repetition were measured and normalized to the maximum peak tetanic tension developed by each sample. A gradual and significant decline from the first to the last experimental session can be clearly noticed (Kruskal-Wallis One Way Analysis of Variance, *P* < 0.05). In details, the force was reduced by ∼50% at the third repetition and by to ∼70% from the fourth repetition, maintaining a plateau until last stimulation session (Figure 2). This decline in peak isometric tetanic tension is a typical indication of the establishment of muscle fatigue (Enoka and Duchateau, 2008; Fitts, 2012).

### Effect of Fatigue on mTOR Phosphorylation

**FIGURE 3:**
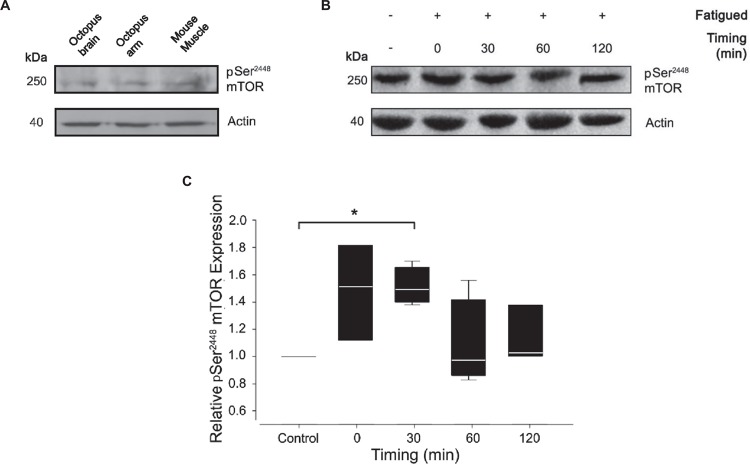
Effect of muscle fatigue induction on mTOR activation. **(A)** pSer^2448^ mTOR and actin antibody validation. Resulting bands were obtained at 250 kDa for pSer^2448^ mTOR and at 42 kDa for actin in lysates from octopus brain, octopus arm and mouse muscle. **(B,C)** Representative experiment **(B)** and quantification **(C)** of western blotting analysis of pSer^2448^ mTOR expression in control and fatigued samples at different timing post RE. Note that the antibody against pSer^2448^ mTOR recognize S2386 in octopus mTOR protein sequence. Data were expressed relative to the control and normalized to β-actin (Kruskal-Wallis One Way Analysis of Variance, Holm-Sidak method, *^∗^P* < 0.05 versus control; *n* = 4 for each experimental group).

To assess the level of mTORC1 activation, we monitored Ser^2386^ mTOR phosphorylation in control and tetanized arms at different time points after the induction of fatigue (0, 30, 60, and 120 min). For Western blotting analysis, we used a polyclonal antibody against residues surrounding Ser^2448^ of human mTOR protein. The antibody specificity for the phosphorylated form of mTOR has been largely confirmed in human, rat and mouse tissues. Based on the extent of protein sequence similarity (see Figure 1), we expected significant cross-reactivity with the homologous proteins in *Octopus vulgaris*. Indeed, the phospho Ser^2448^ mTOR antibody strongly detected a single band at ∼250 kDa in control samples from octopus brain (supraoesophageal mass), octopus arm and mouse muscle (Figure 3A) thus confirming the expression of mTOR in octopus samples. We next performed western blotting analysis on control and RE arm samples (Figure 3B).

Quantification analysis revealed a gradual increase of mTOR phosphorylation reaching a pick 30 minutes from the end of the RE and returning to pre-fatigued values within 60 min (Figure 3C; Mann-Whitney Rank Sum Test; *P* < 0.05 versus control). These variations are the result of a cumulative effect of fatigue in the entire sample composed by muscle and nerve cord. A contribution of nerve cord on the phosphorylation state of mTOR cannot be ruled out; however, given the small volume occupied by the nerve cord (and therefore its contribution to the total lysate), this is considered to be minor.

## Discussion

Cephalopods range greatly in body size and morphology and these differences are displayed in the environment they occupy and in their behavioral pattern. Both habitats and phylogeny have been demonstrated to affect also the metabolism of individual species (Seibel and Carlini, 2001; Seibel, 2007). Moreover, a large body of evidence shows that diet and feeding strategies changes during development, growing, and senescence (Villanueva et al., 2017) and, consequently, the metabolic requirements of a cephalopod are finely regulated alongside each animal life stage and in response to ecosystem fluctuations.

Given the great diversity of cephalopod species, the identification of genes and molecular markers for health and diseases is currently one of the targets of cephalopod European aquaculture.

As muscles represent the major body constituent, studying metabolic pathways may provide important information on animal biology, environmental adaptability and, consequently, on its welfare.

Cephalopod body muscles are mainly striated and composed of uninucleated cells. Although presenting several features typical of cardiac muscles they can be functionally compared to vertebrate skeletal muscles (Zullo et al., 2017) Skeletal muscles play a central role in the maintenance of the body function and integrity, including the generation of movements and the regulation whole-body metabolism (Leone et al., 2005; Izumiya et al., 2008). The control of muscle mass is crucial for mobility, disease resistance, and more in general for the animal wellness. Skeletal muscles have intrinsic adaptability to a wide range of ecological and physiological stimuli such as acute and chronic exercise paradigms (Tsika, 2012). This plasticity is dependent on the ability of muscles to quickly react to external cues, including nutrients, neural activity, growth factors, hormones, and mechanical loading (Bodine, 2006; Frost and Lang, 2007; Sandri, 2008). Indeed, animals commonly experience throughout their life profound changes in muscle metabolism that can bring to muscle hypertrophy (an increase in the size of muscle cells manifested during growing and exercise) or conversely to atrophy (a general decrease in muscle size occurring during aging, systemic disease or injury) (Adams and Bamman, 2012). Cephalopods are similar in those aspects and, furthermore, they manifest two interesting phenomena, namely regeneration and senescence, where relatively rapid but profound modifications of their body occur (Nodl et al., 2015; Imperadore and Fiorito, 2018; Zullo et al., 2018, 2019; Roumbedakis and Guerra, 2019). Therefore, the comprehension of the molecular pathways responsible for muscle mass regulation in this species is strictly important.

The evolutionary conserved mTOR signaling cascade is one major pathway involved in muscle metabolism in vertebrates. mTOR is a highly conserved protein sensing cellular nutrition and energy status and its molecular pathway is related to the muscle resting/stress/exercise state. A number of studies have reported that acute and chronic mechanical loading is sufficient to promote mTORC1 activation, thus regulating skeletal muscle mass. In addition, mTORC1 kinase activity is required for muscle fiber hypertrophy program (Bodine et al., 2001; Rommel et al., 2001; Frost and Lang, 2007; Bentzinger et al., 2008; Sandri, 2008; Risson et al., 2009). mTORC1 has been identified as a master regulator of mRNA translation and protein synthesis and its activation through phosphorylation leads to the inhibition of autophagy-mediated protein degradation (Kim et al., 2011; Sandri, 2013).

Several lines of evidence have shown that mTORC1 signaling have also important functions, both adaptive and maladaptive, in cardiac cells in response to upstream signals, such as IGF-1 cascade, pressure overload and β-adrenergic stimulation.

mTOR expression is required for a correct development of the cardiovascular system in embryo and postnatal life and in the adaptation to stress conditions as shown in animal models of systemic or cardiomyocyte-specific inactivation of mTORC1 (Zhang et al., 2010; Shende et al., 2011).

As said above, octopus muscle cells shared properties typical of cardiac mammalian cells, thus making the octopus arm also extremely interesting in a comparative perspective with cardiac cells.

In fact, it is known that both mechanical stress and workload greatly affects mammalian heart function and efficiency. mTORC1 activation in the heart during chronic stress has also been shown to have detrimental effects, including the induction of pathological hypertrophy and oxidative stress. This is induced by the activation of several hypertrophic signaling cascades and determine an increase in protein synthesis eventually leading to cardiac hypertrophy (Frey et al., 2004). Accordingly, regulated inhibition of mTORC1 activity was shown to reduce cardiovascular damage in response to pressure overload, metabolic cardiomyopathies and aging (Flynn et al., 2013; Wu et al., 2013).

Mechanotransduction is known to be also a mechanism associated with cardiomyopathy through the involvement of sarcomeric Z-disc proteins such as the MLP Family (Frey et al., 2004).

Interestingly, the uninucleated octopus arm muscles are associated with the extracellular membrane through dense body know as peripheral couplings. These are located at the level of the Z line at corresponding locations in adjacent muscle cells and in association with the subsarcolemma cisternae (the octopus muscle sarcoplasmic reticulum) (reviewed in Zullo et al., 2017). Peripheral couplings appears as finger-like processes connecting muscle cells to a collagen matrix (Feinstein et al., 2011).

The nature and function of this structure has not yet been clarified but it seems possible that they participate in muscle coordination and in mechanisms of cell signaling and stretch sensing (Zullo et al., 2017) similarly to what proposed for vertebrates sarcomeres (Luther, 2009).

In addition, vertebrate cardiac and cephalopod striated muscle cells share some physiological properties with cardiac cells (as the presence of Ca^2+^ currents are at the base of spike generation) and few of the genes involved in muscle formation such as NK4, a gene essential for cardiac muscle formation in a number of metazoans, were found to be expressed in cephalopod locomotory muscles (e.g., arm, funnel, mantle; Navet et al., 2008; Bonnaud-Ponticelli and Bassaglia, 2014). Hence, the possibility of comparing both functional and molecular events occurring at the level of single muscle cells in octopus muscles and cardiac cells is of a great advantage.

In this study, we recognized *Octopus vulgaris* mTOR as a member of the phosphatidylinositol kinase family, present in both the brain and muscles as a large multidomain protein. We show that all vertebrate mTOR domains important for the protein activation and function were maintained (for domain annotation analysis see Figure 1). Several conserved phosphorylation sites had been identified in vertebrates: Ser^2448^, Ser^2481^, Thr^2446^, Ser^1261^ (Nave et al., 1999; Peterson et al., 2000; Cheng et al., 2004; Acosta-Jaquez et al., 2009). Among them, Ser^2448^ is specifically involved in the activation of the mTORC1 (Chiang and Abraham, 2005; Copp et al., 2009) whose catalytic function is mediated by the PI3Kinase domain. Interestingly, octopus Ser^2386^ mTOR (corresponding to human and mouse Ser^2448^ mTOR) was found at a conserved location close to the catalytic domain PI3K and FAT C-terminal (FATC) domain, at the COOH-terminus thus suggesting a possible conservation of function of octopus mTORC1 in the control of muscle response to mechanical loading.

To further assess this point we tested a RE protocol on samples of octopus arms and studied the response in term of fatigue induction and phosphorylation at Ser^2386^ mTOR. Octopus arms are mostly muscular and present a peripheral nervous system running in a central position controlling the simultaneous contraction of a relatively large group of muscles (Fossati et al., 2011; Zullo et al., 2011, 2019). We showed that arms undergoing RE rapidly reach and maintain a fatigue state manifested by a reduction of their force at maximal isometric contraction up to ∼70%. Muscle fatigue was accompanied by a gradual, but transient, increase of mTOR phosphorylation reaching a pick 30 min after fatigue induction.

It is conceivable to think that the activation of the mTORC1 pathway here observed is upstream to a cascade of events that may further induce muscle mass increase. Indeed, acute mechanical loading is known to be a major regulator of skeletal muscle mass through mTORC1 signaling cascade, with an increase in mechanical loading resulting in muscle hypertrophy and a decrease resulting in muscle atrophy.

In vertebrates, both skeletal and cardiac muscle are able to adapt to work loads (Russell et al., 2000). Skeletal muscle cells substantially differ in many aspects such as their speed of contraction, intracellular calcium handling, fatigue resistance, as many others. These differences often account for their classification as fast and slow fibers. Relevant to fatigue, this characteristic is known to be greater in fast compared to slow muscles due to factors intrinsic to the motor unit including the pattern of innervation and the set of metabolic changes occurring in the muscle fibers (Allen et al., 2008). Octopus arms are composed by three main muscle types (longitudinal, transverse and oblique muscles) differently arranged within the arm bulk. A detailed study on the fiber identity of each muscle type is not yet available but, based on the current knowledge, cephalopod body muscles seems not to functionally differentiate in slow and fast type as they all express the same types of myosin heavy chains (MHC) (Shaffer and Kier, 2016). Moreover, in the present study all arm muscles were simultaneously stimulated during RE thus all contributing to force and fatigue to various extents. Thus, no direct comparison can be made between the fatigue induction typical of slow and fast mammalian skeletal fibers.

In addition to its involvement during muscle exercise, mTORC1 has also been recently shown to be a key component in the regulation of muscle development and contributes to the formation of new individual muscle cells, a process referred to as hyperplasia (Rion et al., 2019).

This aspect is particularly relevant for the following three considerations: (1) muscle cells in cephalopod body mass are uninucleated, (2) cephalopods have a virtually indeterminate growth, and (3) they manifest high regeneration abilities (Zullo et al., 2017, 2019; Imperadore and Fiorito, 2018). Any increase in body mass may therefore be due to a combination of hypertrophy and hyperplasia, both controlled by mTORC1 signaling pathway. It is noteworthy that cephalopod lifelong increase in body mass cease upon the first mating event. This is accompanied, in both sexes, by the onset of senescence, a short stage of the animal life soon followed by its death (Anderson et al., 2002; Roumbedakis and Guerra, 2019). Hence, we can further suggest mTOR as an indicator of age-related events likewise the occurrence of senescence.

In conclusion, we believe that mTOR can become a versatile tool for measuring the animal health and predictive of diseases in both wild and captive animals. Its use as a marker of wellness may further provide hints for the improvement of animal maintenance procedures of both aquaculture and experimental animals.

## Data Availability

The datasets generated for this study can be found in the GenBank Accession No. KY774846.

## Ethics Statement

The animal study was reviewed and approved by the Local Ethical Committee [OPBA (Organismo Preposto al Benessere degli Animali) of the IRCCS (Istituto di Ricovero e Cura a Carattere Scientifico) Ospedale Policlinico San Martino, Genoa, Italy] and by the Italian Ministry of Health (authorization no. 1111/2016-PR).

## Author Contributions

LZ and AR conceived and designed the research, and interpreted the experimental results. SF performed the first experiments. FM and SG performed the experiments and analyzed the data. LZ carried out the statistical tests. LZ, AR, and FM drafted the manuscript. All authors revised and approved the final version of the manuscript.

## Conflict of Interest Statement

The authors declare that the research was conducted in the absence of any commercial or financial relationships that could be construed as a potential conflict of interest.

## Supplementary Material

TABLE S1 List of primers (forward and reverse) used for octopus mTOR fragments amplification with the relative amplicon lengths.

## References

[B1] Acosta-Jaquez H. A. Keller J. A. Foster K. G. Ekim B. Soliman G. A. Feener E. P. (2009). Site-specific mTOR phosphorylation promotes mTORC1-mediated signaling and cell growth. *Mol. Cell. Biol.* 29 4308–4324. 10.1128/MCB.01665-08 19487463 PMC2715808

[B2] Adams G. R. Bamman M. M. (2012). Characterization and regulation of mechanical loading-induced compensatory muscle hypertrophy. *Compr. Physiol.* 2 2829–2870. 10.1002/cphy.c110066 23720267

[B3] Aguado F. García García B. (2002). Growth and food intake models in *Octopus vulgaris* cuvier/1797: influence of body weight, temperature, sex and diet. *Aquac. Int.* 10 361–377.

[B4] Albertin C. B. Bonnaud L. Brown C. T. Crookes-Goodson W. J. Da Fonseca R. R. Di Cristo C. (2012). Cephalopod genomics: a plan of strategies and organization. *Stand. Genomic Sci.* 7 175–188. 10.4056/sigs.3136559 23451296 PMC3570802

[B5] Allen D. G. Lamb G. D. Westerblad H. (2008). Skeletal muscle fatigue: cellular mechanisms. *Physiol. Rev.* 88 287–332. 10.1152/physrev.00015.2007 18195089

[B6] Anderson R. C. Wood J. B. Byrne R. A. (2002). Octopus senescence: the beginning of the end. *J. Appl. Anim. Welf. Sci.* 5 275–283. 10.1207/s15327604jaws0504_02 16221078

[B7] Bentzinger C. F. Romanino K. Cloëtta D. Lin S. Mascarenhas J. B. Oliveri F. (2008). Skeletal muscle-specific ablation of raptor, but not of rictor, causes metabolic changes and results in muscle dystrophy. *Cell Metab.* 8 411–424. 10.1016/j.cmet.2008.10.002 19046572

[B8] Bodine S. C. (2006). mTOR signaling and the molecular adaptation to resistance exercise. *Med. Sci. Sports Exerc.* 38 1950–1957. 10.1249/01.mss.0000233797.24035.35 17095929

[B9] Bodine S. C. Stitt T. N. Gonzalez M. Kline W. O. Stover G. L. Bauerlein R. (2001). Akt/mTOR pathway is a crucial regulator of skeletal muscle hypertrophy and can prevent muscle atrophy in vivo. *Nat. Cell Biol.* 3 1014–1019. 10.1038/ncb1101-1014 11715023

[B10] Bonnaud-Ponticelli L. Bassaglia Y. (2014). Cephalopod development: what we can learn from differences. *OA Biol.* 2:6.

[B11] Boyle P. Rodhouse P. (2005). *Cephalopods: Ecology and Fisheries.* Oxford: Blackwell Science.

[B12] Cheng S. W. Y. Fryer L. G. D. Carling D. Shepherd P. R. (2004). Thr2446 is a novel mammalian target of rapamycin (mTOR) phosphorylation site regulated by nutrient status. *J. Biol. Chem.* 279 15719–15722. 10.1074/jbc.c300534200 14970221

[B13] Chiang G. G. Abraham R. T. (2005). Phosphorylation of mammalian target of rapamycin (mTOR) at Ser-2448 is mediated by p70S6 kinase. *J. Biol. Chem.* 280 25485–25490. 10.1074/jbc.m501707200 15899889

[B14] Copp J. Manning G. Hunter T. (2009). TORC-specific phosphorylation of mammalian target of rapamycin (mTOR): phospho-Ser2481 is a marker for intact mTOR signaling complex 2. *Cancer Res.* 69 1821–1827. 10.1158/0008-5472.CAN-08-3014 19244117 PMC2652681

[B15] Enoka R. M. Duchateau J. (2008). Muscle fatigue: what, why and how it influences muscle function. *J. Physiol.* 586 11–23. 10.1113/jphysiol.2007.139477 17702815 PMC2375565

[B16] Feinstein N. Nesher N. Hochner B. (2011). Functional morphology of the neuromuscular system of the *Octopus vulgaris* arm. *Vie et Milieu* 61 219–229.

[B17] Fiorito G. Affuso A. Anderson D. B. Basil J. Bonnaud L. Botta G. (2014). Cephalopods in neuroscience: regulations, research and the 3Rs. *Invert. Neurosci.* 14 13–36. 10.1007/s10158-013-0165-x 24385049 PMC3938841

[B18] Fiorito G. Affuso A. Basil J. Cole A. De Girolamo P. D’angelo L. (2015). Guidelines for the care and welfare of cephalopods in research -A consensus based on an initiative by CephRes, FELASA and the boyd group. *Lab. Anim.* 49 1–90. 10.1177/0023677215580006 26354955

[B19] Fitts R. H. (2012). “The muscular system: fatigue processes,” in *ACSM’S Advanced Exercise Physiology*, eds Farrell P. A. Joyner M. J. Caiozzo V. J. (Philadelphia, PA: Wolters Kluwer Health Lippincott Williams &Wilkins).

[B20] Flynn J. M. O’leary M. N. Zambataro C. A. Academia E. C. Presley M. P. Garrett B. J. (2013). Late-life rapamycin treatment reverses age-related heart dysfunction. *Aging Cell* 12 851–862. 10.1111/acel.12109 23734717 PMC4098908

[B21] Fossati S. M. Benfenati F. Zullo L. (2011). Morphological characterization of the *Octopus vulgaris* arm. *Vie et Milieu* 61 197–201.

[B22] Frey N. Katus Hugo A. Olson Eric N. Hill Joseph A. (2004). Hypertrophy of the heart. *Circulation* 109 1580–1589. 15066961 10.1161/01.CIR.0000120390.68287.BB

[B23] Frost R. A. Lang C. H. (2007). Protein kinase B/Akt: a nexus of growth factor and cytokine signaling in determining muscle mass. *J. Appl. Physiol.* 103 378–387. 10.1152/japplphysiol.00089.2007 17332274

[B24] García García B. Cerezo Valverde J. (2006). Optimal proportions of crabs and fish in diet for common octopus (*Octopus vulgaris*) ongrowing. *Aquaculture* 253 502–511. 10.1016/j.aquaculture.2005.04.055

[B25] Garrett S. Rosenthal J. J. (2012). RNA editing underlies temperature adaptation in K+ channels from polar octopuses. *Science* 335 848–851. 10.1126/science.1212795 22223739 PMC4219319

[B26] Iglesias J. Sanchez F. J. Otero J. Moxica C. (2000). “Ongrowing, reproduction and larvae rearing of octopus (*Octopus vulgaris* c.), a new candidate for aquaculture in Galicia (NW Spain),” in *Proceedings of the workshop on New Species for Aquaculture*, Faro Portugal, 53–55.

[B27] Imperadore P. Fiorito G. (2018). Cephalopod tissue regeneration: consolidating over a century of knowledge. *Front. Physiol.* 9:593. 10.3389/fphys.2018.00593 29875692 PMC5974545

[B28] Izumiya Y. Hopkins T. Morris C. Sato K. Zeng L. Viereck J. (2008). Fast/glycolytic muscle fiber growth reduces fat mass and improves metabolic parameters in obese mice. *Cell Metab.* 7 159–172. 10.1016/j.cmet.2007.11.003 18249175 PMC2828690

[B29] Kim J. Kundu M. Viollet B. Guan K.-L. (2011). AMPK and mTOR regulate autophagy through direct phosphorylation of Ulk1. *Nat. Cell Biol.* 13 132–141. 10.1038/ncb2152 21258367 PMC3987946

[B30] Leone T. C. Lehman J. J. Finck B. N. Schaeffer P. J. Wende A. R. Boudina S. (2005). PGC-1α deficiency causes multi-system energy metabolic derangements: muscle dysfunction, abnormal weight control and hepatic steatosis. *PLoS Biol.* 3:e101. 10.1371/journal.pbio.0030101 15760270 PMC1064854

[B31] Luther P. K. (2009). The vertebrate muscle Z-disc: sarcomere anchor for structure and signalling. *J. Muscle Res. Cell Motil.* 30 171–185. 10.1007/s10974-009-9189-6 19830582 PMC2799012

[B32] Milligan B. Curtin N. Bone Q. (1997). Contractile properties of obliquely striated muscle from the mantle of squid (*Alloteuthis subulata*) and cuttlefish (*Sepia officinalis*). *J. Exp. Biol.* 200 2425–2436. 9320349 10.1242/jeb.200.18.2425

[B33] Nakajima R. Shigeno S. Zullo L. De Sio F. Schmidt M. R. (2018). Cephalopods between science, art, and engineering: a contemporary synthesis. *Front. Commun.* 3:20 10.3389/fcomm.2018.00020

[B34] Nave B. T. Ouwens M. Withers D. J. Alessi D. R. Shepherd P. R. (1999). Mammalian target of rapamycin is a direct target for protein kinase B: identification of a convergence point for opposing effects of insulin and amino-acid deficiency on protein translation. *Biochem. J.* 344(Pt 2), 427–431. 10.1042/bj3440427 10567225 PMC1220660

[B35] Navet S. Bassaglia Y. Baratte S. Martin M. Bonnaud L. (2008). Somatic muscle development in *Sepia officinalis* (*cephalopoda* - *mollusca*): a new role for NK4. *Dev. Dyn.* 237 1944–1951. 10.1002/dvdy.21614 18570246

[B36] Nodl M. T. Fossati S. M. Domingues P. Sanchez F. J. Zullo L. (2015). The making of an octopus arm. *Evodevo* 6:19. 10.1186/s13227-015-0012-8 26052417 PMC4458049

[B37] Peterson R. T. Beal P. A. Comb M. J. Schreiber S. L. (2000). FKBP12-rapamycin-associated protein (FRAP) autophosphorylates at serine 2481 under translationally repressive conditions. *J. Biol. Chem.* 275 7416–7423. 10.1074/jbc.275.10.7416 10702316

[B38] Rion N. Castets P. Lin S. Enderle L. Reinhard J. R. Eickhorst C. (2019). mTOR controls embryonic and adult myogenesis via mTORC1. *Development* 146:dev172460. 10.1242/dev.172460 30872276

[B39] Risson V. Mazelin L. Roceri M. Sanchez H. Moncollin V. Corneloup C. (2009). Muscle inactivation of mTOR causes metabolic and dystrophin defects leading to severe myopathy. *J. Cell Biol.* 187 859–874. 10.1083/jcb.200903131 20008564 PMC2806319

[B40] Rocchi A. Milioto C. Parodi S. Armirotti A. Borgia D. Pellegrini M. (2016). Glycolytic-to-oxidative fiber-type switch and mTOR signaling activation are early-onset features of SBMA muscle modified by high-fat diet. *Acta Neuropathol.* 132 127–144. 10.1007/s00401-016-1550-4 26971100 PMC4911374

[B41] Rommel C. Bodine S. C. Clarke B. A. Rossman R. Nunez L. Stitt T. N. (2001). Mediation of IGF-1-induced skeletal myotube hypertrophy by PI(3)K/Akt/mTOR and PI(3)K/Akt/GSK3 pathways. *Nat. Cell Biol.* 3 1009–1013. 10.1038/ncb1101-1009 11715022

[B42] Roumbedakis K. Guerra A. (2019). “Chapter 16_cephalopod senescence and parasitology,” in *Handbook of Pathogens and Diseases in Cephalopods*, eds Gestal C. (Berlin: Springer).

[B43] Russell B. Motlagh D. Ashley W. (2000). Form follows function: how muscle shape is regulated by work. *J. Appl. Physiol.* 88 1127–1132. 10.1152/jappl.2000.88.3.1127 10710412

[B44] Sandri M. (2008). Signaling in muscle atrophy and hypertrophy. *Physiology* 23 160–170. 10.1152/physiol.00041.2007 18556469

[B45] Sandri M. (2013). Protein breakdown in muscle wasting: role of autophagy-lysosome and ubiquitin-proteasome. *Int. J. Biochem. Cell Biol.* 45 2121–2129. 10.1016/j.biocel.2013.04.023 23665154 PMC3775123

[B46] Saxton R. A. Sabatini D. M. (2017). mTOR signaling in growth, metabolism, and disease. *Cell* 168 960–976. 10.1016/j.cell.2017.02.004 28283069 PMC5394987

[B47] Schiaffino S. Dyar K. A. Ciciliot S. Blaauw B. Sandri M. (2013). Mechanisms regulating skeletal muscle growth and atrophy. *FEBS J.* 280 4294–4314. 10.1111/febs.12253 23517348

[B48] Seibel B. A. (2007). On the depth and scale of metabolic rate variation: scaling of oxygen consumption rates and enzymatic activity in the Class Cephalopoda (Mollusca). *J. Exp. Biol.* 210 1–11. 10.1242/jeb.02588 17170143

[B49] Seibel B. A. Carlini D. B. (2001). Metabolism of pelagic cephalopods as a function of habitat depth: a reanalysis using phylogenetically independent contrasts. *Biol. Bull.* 201 1–5. 10.2307/1543519 11526057

[B50] Shaffer J. F. Kier W. M. (2016). Tuning of shortening speed in coleoid cephalopod muscle: no evidence for tissue-specific muscle myosin heavy chain isoforms. *Invert. Biol.* 135 3–12. 10.1111/ivb.12111 26997860 PMC4795958

[B51] Shende P. Plaisance I. Morandi C. Pellieux C. Berthonneche C. Zorzato F. (2011). Cardiac raptor ablation impairs adaptive hypertrophy, alters metabolic gene expression, and causes heart failure in mice. *Circulation* 123 1073–1082. 10.1161/CIRCULATIONAHA.110.977066 21357822

[B52] Thompson J. T. Shelton R. M. Kier W. M. (2014). The length–force behavior and operating length range of squid muscle vary as a function of position in the mantle wall. *J. Exp. Biol.* 217 2181–2192. 10.1242/jeb.083907 24675565

[B53] Tsika R. (2012). “The muscular system: the control of muscle mass,” in *ACSM’s Advanced Exercise Physiology*, 2nd Edn, eds Farrell P. A. Caiozzo M. J. Joyner V. J. (Baltimore, MD: Lippincott Williams & Wilkins).

[B54] Villanueva R. Perricone V. Fiorito G. (2017). Cephalopods as predators: a short journey among behavioral flexibilities. adaptions, and feeding habits. *Front. Physiol.* 8:598. 10.3389/fphys.2017.00598 28861006 PMC5563153

[B55] Wu X. Cao Y. Nie J. Liu H. Lu S. Hu X. (2013). Genetic and pharmacological inhibition of Rheb1-mTORC1 signaling exerts cardioprotection against adverse cardiac remodeling in mice. *Am. J. Pathol.* 182 2005–2014. 10.1016/j.ajpath.2013.02.012 23567640

[B56] Yoon M.-S. (2017). mTOR as a key regulator in maintaining skeletal muscle mass. *Front. Physiol.* 8:788. 10.3389/fphys.2017.00788 29089899 PMC5650960

[B57] Zhang D. Contu R. Latronico M. V. G. Zhang J. Rizzi R. Catalucci D. (2010). MTORC1 regulates cardiac function and myocyte survival through 4E-BP1 inhibition in mice. *J. Clin. Invest.* 120 2805–2816. 10.1172/JCI43008 20644257 PMC2912201

[B58] Zoncu R. Efeyan A. Sabatini D. (2011). mTOR: from growth signal integration to cancer, diabetes and ageing. *Nat. Rev. Mol. Cell Biol.* 12 21–35. 10.1038/nrm3025 21157483 PMC3390257

[B59] Zullo L. Buschiazzo A. Massollo M. Riondato M. Democrito A. Marini C. (2018). Small-animal (18)F-FDG PET for research on *Octopus vulgaris*: applications and future directions in invertebrate neuroscience and tissue regeneration. *J. Nucl. Med.* 59 1302–1307. 10.2967/jnumed.117.205393 29523626

[B60] Zullo L. Eichenstein H. Maiole F. Hochner B. (2019). Motor control pathways in the nervous system of *Octopus vulgaris* arm. *J. Comp. Physiol. A Neuroethol. Sens. Neural Behav. Physiol*. 205 271–279. 10.1007/s00359-019-01332-6 30919046 PMC6478645

[B61] Zullo L. Fossati S. M. Benfenati F. (2011). Transmission of sensory responses in the peripheral nervous system of the arm of *Octopus vulgaris*. *Vie et Milieu* 61 197–201.

[B62] Zullo L. Fossati S. M. Imperadore P. Nodl M. T. (2017). Molecular determinants of cephalopod muscles and their implication in muscle regeneration. *Front. Cell Dev. Biol.* 5:53. 10.3389/fcell.2017.00053 28555185 PMC5430041

